# Significant lifespan difference between primary open-angle glaucoma and pseudoexfoliation glaucoma

**DOI:** 10.1016/j.heliyon.2021.e06421

**Published:** 2021-03-13

**Authors:** Jon Klokk Slettedal, Leiv Sandvik, Amund Ringvold

**Affiliations:** a Institute of Clinical Medicine, University of Oslo, Norway; b Department of Ophthalmology, Oslo University Hospital, Norway; c Oslo Center for Biostatistics and Epidemiology, Oslo University Hospital, Norway

**Keywords:** Primary open-angle glaucoma, Pseudoexfoliation glaucoma, Pseudoexfoliation syndrome, Lifespan, Systemic diseases

## Abstract

**Purpose:**

Open-angle glaucoma (OAG) is a collective term for various subgroups of glaucoma of which primary open-angle glaucoma (POAG) and pseudoexfoliation glaucoma (PEG) are the most common. There is increasing evidence that both conditions have systemic ramifications. We wanted to examine to what extent lifespan and cause of death are influenced by POAG, pseudoexfoliation syndrome (PES), and PEG.

**Materials and methods:**

Of 1864 people who underwent an eye examination in 1985–86, the presence of PES and/or glaucoma, along with date and cause of death were recorded. Based on information from the National Death Registry, the individuals were classified into the following groups of systemic diseases regarded as causing death: Cardiovascular disease (with two subgroups), cerebrovascular disease and neoplasms.

**Results:**

All 1864 persons were followed to death, up to 30 years after examination. No difference in lifespan was observed when comparing OAG (i.e. POAG and PEG together) with the rest of the population. When adjusting for gender and age at inclusion, patients with POAG showed a reduced lifespan in the cardiovascular death group (2.44 years, p = 0.043). When comparing lifespan in the neoplastic group in the glaucoma patients, POAG and PEG, directly against each other, a mean age difference of 6.87 years (p = 0.017) was found.

**Conclusions:**

POAG patients showed reduced lifespan due to neoplasia and cardiovascular disease. Persons with PES and PEG did not show these lifespan reductions. Our main conclusion is that POAG and PEG, the two main OAG subgroups, are very different disease entities both from an ocular and a systemic point of view.

## 1 Introduction

Several previous studies, including a meta-analysis, could not demonstrate any association between OAG and survival [1, 2]. It is important to note that the term OAG includes various subgroups, of which, on a worldwide basis, POAG and PEG are the most common. PEG has a pre-stage called ocular PES which is characterized by the presence of grey deposits on the anterior lens surface and along the pupillary border. This condition is regarded as a warning symptom because it may gradually transform into glaucoma, i.e. PEG. In contrast to PEG, POAG occurs without any presage. Both of the entities typically show progressive visual field defects and increased intraocular pressure (IOP), although normotensive cases are not uncommon.

To what extent POAG and PEG share pathogenetic mechanisms is still open for debate. POAG eyes have no visible abnormality in the anterior chamber angle by gonioscopy. Both subgroups show abnormal extracellular material in the trabecular meshwork. However, the aberrant matrix in the two conditions reveals marked morphological differences: The amount of sheath-derived material (“plaque material”) is significantly greater in the trabecular area of POAG compared to PEG [3], whereas the outflow area in PEG-eyes is dominated by typical pseudoexfoliation aggregates [4, 5, 6]. This implies that, despite both PEG and POAG showing intraocular connective tissue changes contributing to increased aqueous outflow resistance and elevated IOP, they are different entities.

Regarding systemic associations with POAG, a major review concluded that this condition is not just a process involving the visual system, but more likely the manifestation of generalized systemic dysfunction [7]. More specifically, POAG is now described as an optic neuropathy with focus on neurotrophins, compromized axonal transport, and oxidative stress [8], and recently it was suggested that POAG and ischemic heart disease may have pathogenetic similarities [9]. In addition, increased risk of all-cause and cardiovascular mortality has been reported for patients with this type of glaucoma [10, 11].

In contrast to patients with POAG, people with PEG (and PES) show morphological changes in vascular and connective tissues throughout the body [12, 13, 14, 15]. Some studies claim that patients with PES have increased vascular morbidity [16, 17, 18] or that there is a linkage to cardiovascular and cerebrovascular disease [19, 20]. A recent meta-analysis concluded that the overall literature suggests that pseudoexfoliation is associated with an increased risk of cardiovascular disease [21]. However, in other studies, all-cause mortality was not affected [22, 23], and may even be reduced in PES-positive persons [24]. Indeed, lifespan was not statistically different in persons with and without PES in a 30-year follow-up study [25]. Furthermore, in patients with PES, the lifespan reduction that would be expected in patients dying due to neoplasia was not present [26].

Thus, the possible systemic associations of POAG and PES are still unclear and it is reasonable to conclude that a mortality and/or lifespan change in these diseases cannot be excluded. Therefore, the aim of the present study is to evaluate the impact on lifespan and cause of death in patients suffering from POAG and PES.

## 2 Material and methods

This study is based on data from an epidemiological survey conducted in the three municipalities Hitra (Hi), Holtålen (Ho), Rennebu (Re) in the county of Sør-Trøndelag, Norway in 1985–86 [27]. All inhabitants above 64 years of age were invited (2109 persons), 1888 (1018 women, 870 men, ethnic Norwegians) were examined, while 221 chose not to participate. Informed consent was obtained according to the Helsinki declaration.

A temporary test center was established in each municipality and screening was performed by an ophthalmologist according to the following schedule: Intraocular pressure (IOP) was measured with a Goldmann applanation device after 0.5% proxymetacaine anesthesia, followed by pupil dilation with 0.5% tropicamide. Subsequently, conventional slit-lamp examination was performed to check for PE-material and optic disc excavation, including vertical cup/disc-ratio judged by direct ophthalmoscopy. Due to various ocular conditions (corneal opacities, enucleation etc.) 43 persons had only one eye examined. The presence of PES, PEG, and POAG are all irreversible diagnoses once they have been confirmed.

Individuals with IOP ≥25 mmHg and/or glaucomatous cupping were referred to the University Eye Department in Trondheim for registration of visual acuity, refraction, IOP, and visual field examination (Humphrey, “Armaly Full Field, quantify defects”). Some patients, already under glaucomatous treatment, preferred to attend their own ophthalmologist, from whom the information was collected. These cases had also visual field registration, though with methods differing from the hospital-based set-up.

A person was classified as having glaucoma when 2 of the following 3 criteria were present [28]: 1) IOP ≥25 mm Hg (mean value from 3 measurements), 2) Vertical cup/disc-ratio ≥ 0.8, 3) Glaucomatous field defects, i.e. nasal step, arcuate scotoma or more advanced defects. As defects were considered areas with minimum three adjacent test points showing depressions of at least 4 dB.

The following abbreviations have been used:

PES^Total^ = all cases with PES regardless of the presence of glaucoma or not (n = 317)
PES^Alone^ = PES cases without glaucoma (n = 228)
PEG = pseudoexfoliation cases with glaucoma (n = 89)
POAG = glaucoma without pseudoexfoliation (n = 63)
OAG = POAG and PEG combined (n = 152)

Ocular hypertension (OHT) was defined as IOP ≥25 mm Hg in otherwise normal eyes, a definition not uncommon at that time. Persons showing PES in one or both eyes were registered as PES-positive. If only one eye could be evaluated, and found PES-negative, the case was grouped as undefined. Calculations on POAG and PEG did not include OHT, angle-closure glaucoma, neovascular glaucoma, and uveitic glaucoma. Glaucoma in one eye only was grouped as a glaucoma case.

Following approval from the Regional Ethical Committee, mortality information, including death date and cause(s) of death, was obtained from The Norwegian Cause of Death Registry. Every death in Norway is reviewed by a medical doctor who examines the deceased person and fills in a death certificate with compulsory diagnostic coding. Neoplasia cases are routinely examined with either biopsy or autopsy. Autopsy is otherwise performed in the minority of cases, usually when death is unexpected, or the cause is uncertain. As of 01.01.16, of the 1888 initially examined individuals, 13 were still alive, and 11 persons were recorded as deceased without any diagnostic coding. Thus, 1864 participants (98.7%) remained for analysis.

The cause of death was coded according to WHOs International Statistical Classification of Diseases and Related Health Problems (ICD) version 8 (used 1969–85), 9 (used 1986–95), and 10 (used from 1996). 574 persons (30.1%) had a single diagnosis indicating cause of death, and the remaining 1290 persons had one diagnosis indicating the primary cause of death, and in addition one or more (up to a maximum of 9) diagnoses that were considered contributing to death. Based on the ICD-coding, the deceased were classified in the following groups according to the cause of death: Cardiovascular disease (further divided into two subgroups), cerebrovascular disease, and neoplasms. Lifespan in various subgroups are compared with lifespan in the remaining population.

### 2.1 Statistical analysis

In order to compare mean lifespan in two subgroups, and adjust for age at inclusion and gender, linear regression analysis was performed with lifespan as the dependent variable, and age at inclusion, gender and cause of death as independent variables. A significance level of 5% was used. The statistical analysis was performed using IBM-SPSS version 20 software.

## 3 Results

PES^Total^ prevalence in the three municipalities Hi, Ho, and Re was 10.2 %, 21.0 %, and 19.6 %, respectively. The municipalities Ho + Re showed very similar prevalence, and accordingly, the possible impact of PES per se on lifespan could therefore be estimated by comparing numbers from Hi versus Ho + Re: Lifespan in Hi was 85.6 (SD 6.7) years, versus 85.7 (SD 6.7) years in Ho + Re (p = 0.714). There was no difference in sex distribution between the two groups (p = 0.639).

With respect to glaucoma, the data was analyzed for differences in lifespan and cause of death in different categories in PES-negative persons versus POAG, PES^Total^, and PEG, respectively. Of the 1864 (1003 females, 861 males) deceased with registered cause of death, 17.0 % showed ocular PES at examination in 1985–86, against 17.9 % in the neoplasm group (397 persons). In total, 28.1 % of the PES^Total^ cases were recorded with PEG at baseline. 152 persons (8.15 %) had glaucoma in the total population. Additional core information is presented in Table 1.

**Table 1:** Overview showing lifespan of all cases and subgroups.

	All cases		POAG		PES^Total^		PES^Alone^		PEG	
		♂/♀		♂/♀		♂/♀		♂/♀		♂/♀
Groups	1864 (100%)	861/1003	63 (3.4%)	26/37	317 (17.0%)	125/192	228 (12.2%)	81/147	89 (4.8%)	44/45
Mean lifespan (years)	85.7	84.3/86.8 (p < 0.001)	85.0	83.0/86.4 (p = 0.036)	87.2	86.6/87.6 (p = 0.187)	86.8	86.3/87.0 (p = 0.364)	88.3	87.2/89.3 (p = 0.153)

### 3.1 Comparison of lifespan

The following groups of relevant diseases were evaluated: Cardiovascular disease with subgroups a) acute myocardial infarction and b) other cardiovascular disease, cerebrovascular disease, and neoplasms. Due to low numbers, other conditions (systemic hypertension, diabetes mellitus, chronic obstructive lung disease, Parkinson's disease, aortic aneurysm, amyloidosis, and trauma) have not been analyzed. No change in lifespan was observed in any of the categories when testing OAG (i.e. POAG and PEG together) against the rest of the population within each single category (Table 2). However, by testing POAG, PES^Total^, PES^Alone^, and PEG individually, differences were seen. The cardiovascular death group showed reduced lifespan in the POAG patients (Table 2, p = 0.043). Furthermore, both PES^Total^ and PES^Alone^ lived significantly longer than the control population in the neoplasm category (Table 2, p = 0.011 and p = 0.026, respectively).

**Table 2:** Lifespan difference (years) in diagnostic groups (according to cause of death) compared to the rest of the population in each category, adjusted for gender and age at inclusion. p-value in brackets. n = number of persons.

Diagnostic group	No of persons	OAG (=POAG and PEG)	POAG	PES^Total^	PES^Alone^	PEG
All cases	1864	+0.139 (0.784) (n = 152)	−1.380 (0.075) (n = 63)	+0.346 (0.354) (n = 317)	−0.490 (0.908) (n = 228)	+1.208 (0.070) (n = 89)
Cardiovascular, total	685	−0.505 (0.552) (n = 58)	−2.440 (0.043) (n = 27)	−0.351 (0.592) (n = 111)	−0.990 (0.182) (n = 80)	+1.262 (0.267) (n = 31)
*Acute myoc. inf.*	484	−0.641 (0.507) (n = 42)	−2.444 (0.072) (n = 20)	−0.503 (0.509) (n = 75)	−1.154 (0.188) (n = 53)	+1.094 (0.404) (n = 22)
*Other cardiovasc.*	201	+0.860 (0.560) (n = 16)	−0.791 (0.714) (n = 7)	+0.209 (0.845) (n = 36)	−0.538 (0.650) (n = 27)	+2.069 (0.280) (n = 9)
Cerebrovascular	240	+0.803 (0.546) (n = 22)	−0.660 (0.743) (n = 9)	−0.381 (0.704) (n = 43)	÷1.354 (0.245) (n = 30)	+1.769 (0.291) (n = 13)
Neoplasms	397	−0.137 (0.902) (n = 29)	−3.674 (0.069) (n = 8)	+1.940 (0.011) (n = 71)	+1.962 (0.026) (n = 50)	+1.312 (0.310) (n = 21)
Non-neoplasms	1467	+0.130 (0.818) (n = 123)	−1.261 (0.126) (n = 55)	+0.014 (0.973) (n = 246)	−0.510 (0.291) (n = 178)	+1.265 (0.091) (n = 68)

**Figure 1:**
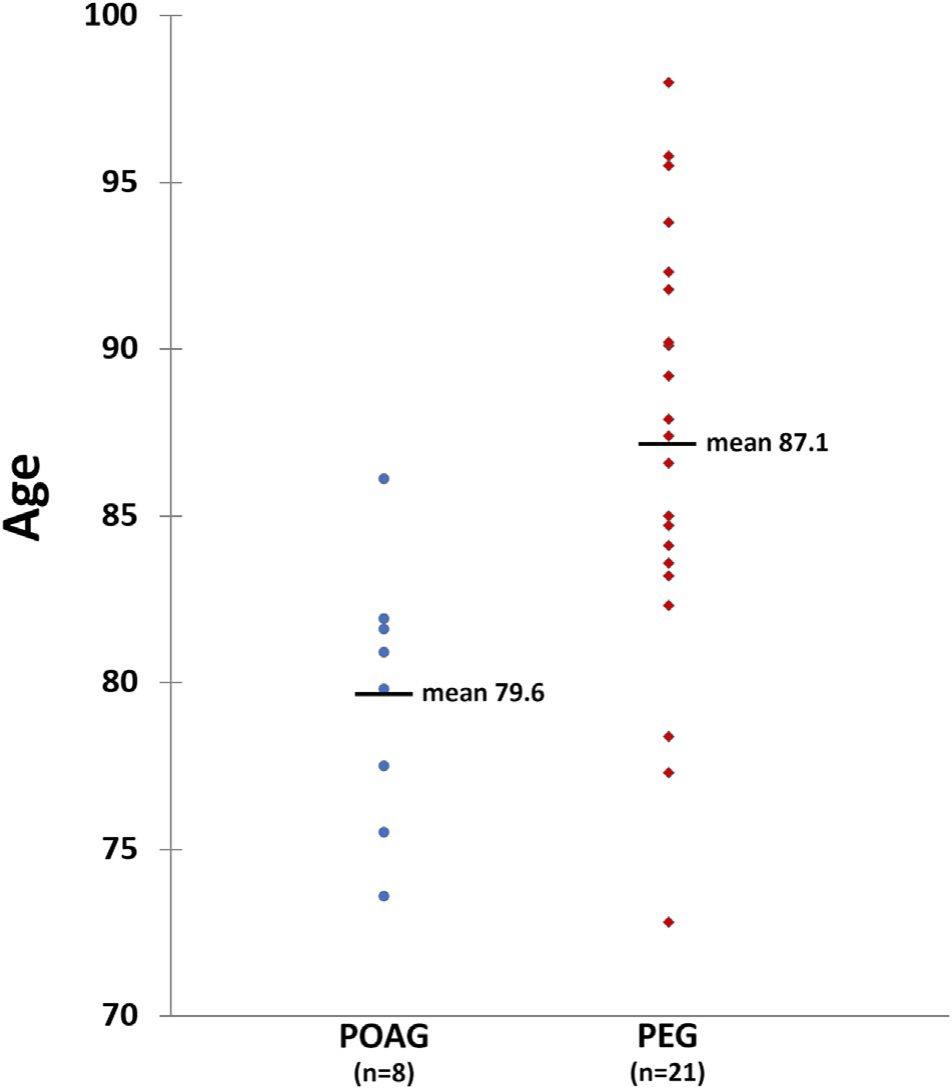
Life span of all patients with OAG and neoplasm are shown, i.e. 8 patients with POAG and 21 with PEG. Mean lifespan is 79.6 years and 87.1 years, respectively (difference 7.5 years). After adjustment for gender and age at inclusion the difference is 6.9 years.

The most striking observations occurred in the neoplasia category: Comparison of lifespan in POAG versus PEG, and POAG versus PES^Total^ showed mean differences of 4.99 years (see Table 2; -3.67 and +1,31 combined) and 5.61 years (-3.67 and +1.94), respectively. Comparing lifespan in the glaucoma patients, POAG and PEG, against each other results in a mean age difference of 6.87 years (p = 0.017). A separate plot shows the actual lifespan of all 29 glaucoma patients who died due to a neoplasm (Figure 1), and the differences in lifespan between POAG and PEG are clearly seen.

### 3.2 Comparison of cause of death

The number of persons in each cause of death group and prevalence of POAG, PES, and PEG are presented in Table 3. No statistically significant differences were observed between each of these subgroups and the respective reference population consisting of the remaining persons. It should be noted that the number of POAG patients with cardiovascular death diagnosis was not increased (Table 3, p = 0.306). The prevalence of neoplasia was similar in the PES-negative (n = 1547) and the PES-positive subgroups (n = 317), 21.1 % and 22.4 %, respectively (p = 0.862). In total, 63 POAG and 89 PEG cases were observed, including 8 and 21 with neoplasms, respectively. However, the neoplasia category contained 5.3% PEG, against 4.6% in the non-neoplasia group (p = 0.588). Comparable figures for POAG were 2.0% and 3.7%, respectively (p = 0.090).

**Table 3:** Cause of death in POAG, PES^Total^, and PEG compared to the rest of the population, adjusted for gender and age at inclusion. No significant differences were observed. n = number of persons.

Diagnostic group	All cases	PES-negative	POAG		PES^Total^		PEG	
			n (%)	p-value	n (%)	p-value	n (%)	p-value
All cases	1864	1547	63 (100)		317 (100)		89 (100)	
Cardiovascular, total	685	574	27 (42.9)	0.306	111 (35.0)	0.482	31 (34.8)	0.701
*Acute myoc. inf.*	484	409	20 (31.7)	0.287	75 (23.7)	0.304	22 (24.7)	0.783
*Other cardiovasc.*	201	165	7 (11.1)	0.932	36 (10.8)	0.718	9 (10.1)	0.834
Cerebrovascular	240	197	9 (14.3)	0.734	43 (13.6)	0.688	13 (14.6)	0.617
Neoplasms	397	326	8 (12.7)	0.090	71 (22.4)	0.600	21 (23.6)	0.588

## 4 Discussion

The aim of the present study was to evaluate the relation between lifespan and cause of death in patients with coexisting POAG or PEG. 1864 persons above 64 years were examined in 1985–86, and thereafter followed to death, up to 30 years later. This was a relatively complete cohort (90% of the total population in the participating areas) and there was minimal dropout. Thus, these observations are not restricted to a limited group with early death, but rather represents the actual lifespan in the total population. Furthermore, apart from presence or absence of PES, the same definition of glaucoma has been used for both PEG and POAG [28], which can be an issue in large-cohort retrospective studies. Finally, the diagnostic precision in the neoplasm group is high, due to hospitalization of these patients with biopsy verification of the diagnosis.

In a previous study we showed that lifespan is not statistically different in persons with or without PES in the same cohort [25]. This is in keeping with our present observation of no difference in lifespan (p = 0.714) between a pooled group of the two municipalities (Ho and Re) versus Hi, despite marked difference in PES prevalence (21.0 % and 19.6 % in Ho and Re, respectively versus 10.2 % in Hi). Together these figures confirm that PES per se (i.e. without additional factors) does not influence lifespan.

The relationship between OAG and mortality is still a matter of controversy. Increased mortality due to cardiovascular disease has been reported in OAG patients [11]. However, recent reports have found no association between OAG and all-cause mortality [1, 2]. Our observations are in line with the latter conclusion as no lifespan changes in OAG (i.e. POAG and PEG combined) were observed in any of the diagnostic categories when compared to the rest of the population, in the respective groups (Table 2).

### 4.1 POAG evaluation

When dividing OAG into the subgroups POAG and PEG (Table 2), a consistent relationship became apparent: PEG patients tended to live longer (though not statistically significant) than the non-PEG control population in all diagnostic categories, in contrast to comparable results for POAG showing a reduced lifespan. The lifespan in POAG patients was significantly less compared to the control group in the cardiovascular category (Table 2, p = 0.043). It should be noted that there are not more persons dying from cardiovascular disease in the POAG group then expected, however, persons with coexisting cardiovascular disease and POAG showed a reduced lifespan. In addition, a marked lifespan reduction in POAG patients was seen in the neoplasm group, though not statistically significant (Table 2, p = 0.069, n = 8). One by one or together, these factors appear to contribute to reduced lifespan in POAG patients.

### 4.2 PES^Total^ evaluation

Several reports claim there is strong evidence that PES is significantly associated with both cardiovascular and cerebrovascular disease [20]. A recent meta-analysis based on 18 selected reports concluded that ischemic heart disease, in general, had a statistically significant association with PES, whereas myocardial infarction, chronic ischemic heart disease, angina, and hypertension did not show such a correlation [19]. Furthermore, based on 6046 PES-positive cases, various stages of ischemic heart disease were significantly associated with ocular PES [29]. One recent study found increased cardiovascular disease, as well as increased risk of pulmonary and urogenital disorders among PES-positive persons [30]. This is contradicted by other papers that failed to show any link between cardiovascular disease related mortality with PES [24, 26]. Indeed, the present study did not indicate any difference in lifespan or cause of death diagnoses in the PES^Total^ group versus the control in cardiovascular disease (Table 2, p = 0.592; Table 3, p = 0.482, respectively). Furthermore, overall lifespan was equal in the inter-municipal comparison [Hi, 85.6 years versus Ho and Re combined, 85.7 years (p = 0.714)], despite PES prevalence figures of 10.2% and 20.34% respectively. Given the level of contradictory results, definitive conclusions are difficult to draw.

### 4.3 PEG evaluation

Lifespan comparable with controls was observed for PEG in all diagnostic categories (Table 2). Additionally, the cause of death diagnoses was within normal range in every diagnostic category (Table 3). Furthering the findings of PES^Total^ not having a detrimental effect on lifespan, these results do not elucidate any link between PEG and cardiovascular disease either. However, cardiovascular morbidity analysis is not possible within the parameters of this study.

### 4.4 PES^Total^ and PES^Alone^ evaluation

PES^Total^ and PES^Alone^ showed normal lifespan in all categories compared to the rest of the population, except for the neoplasia diagnostic group (Table 2). As could be expected, the death due to neoplasia group showed a reduced lifespan. However, and somewhat surprisingly, in a previous paper, we demonstrated that patients who had both neoplasia and PES showed a normal lifespan [26]. Consequently, in the neoplasia category in Table 2, normal lifespan is represented by the PES^Total^ and PES^Alone^ values, versus the reference population showing reduced lifespan due to neoplasia. Interestingly, this normalisation is not seen in the PEG-column (Table 2, p = 0.310), perhaps due to low numbers (n = 21).

### 4.5 POAG and PEG evaluation

When comparing the lifespan of POAG and PEG groups, remarkable differences are observed in all categories (Table 2). The most significant difference is seen in the neoplasm category where POAG patients lived 4.99 years less than PEG patients, increasing to 5.61 years when compared to PES^Total^. This substantial difference in the neoplasia category is likely due to three factors: 1) lifespan of PEG patients is normal in the neoplasia category, 2) POAG patients have reduced lifespan due to neoplasia, 3) in addition, there is a significant lifespan reduction in POAG patients due to cardiovascular disease. The last phenomenon is shown in Table 2 (p = 0.043). Interestingly, a Finnish study found increased mortality in POAG patients compared to PEG patients [31]. Cardiovascular disorders in POAG patients have also been highlighted by other authors [18, 19, 29]. On the other hand, there was not an increased incidence of cardiovascular death linked to POAG in the present study (Table 3, p = 0.306). Some caution should be observed when drawing conclusions, however, because of the small numbers involved (POAG n = 8, PEG n = 21).

A shortcoming of our study is the fact that the ocular status was established in 1985–86, whereas the death diagnoses were recorded during the following 30 years. As the prevalence of POAG, PES, and PEG all increase with age, their numbers are underestimated when compared to rates at death. However, as they all are underestimated, this bias is at least minimized when mutual comparisons are performed. It should also be noted that, unlike with the neoplasm group that was confirmed with autopsy or biopsy, there is some uncertainty linked to the other death diagnoses as these were given by a medical doctor on site.

In conclusion, patients who have PEG had normal lifespans, whilst patients with POAG had a reduced lifespan. A statistically significant lifespan difference was observed between POAG and PEG in the neoplasm death group, with POAG patients’ lifespan being shorter by 4.99 years. A possible explanation could be that neoplasia reduces lifespan in general, except for patients with PES perhaps suggesting a protective element. In addition, POAG patients showed reduced lifespan in the cardiovascular category. Consequently, POAG and PEG, the two OAG subgroups, are very different disease entities both from an ocular and a systemic point of view.

## Declarations

### Author contribution statement

Jon Klokk Slettedal and Amund Ringvold: Conceived and designed the experiments; Performed the experiments; Analyzed and interpreted the data; Contributed reagents, materials, analysis tools or data; Wrote the paper.

Leiv Sandvik: Conceived and designed the experiments; Performed the experiments; Analyzed and interpreted the data; Contributed reagents, materials, analysis tools or data.

### Funding statement

This work was supported by The Norwegian Society of the Blind and Partially Sighted and the Raagholt Research Foundation.

### Data availability statement

Data will be made available on request.

### Declaration of interests statement

The authors declare no conflict of interest.

### Additional information

No additional information is available for this paper.

## References

[1] Akbari M., Akbari S., Pasquale L.R. (2009). The association of primary open-angle glaucoma with mortality. A meta-analysis of observational studies. Arch. Ophthalmol..

[2] Sundquist J., Ekström C. (2018). Open-angle glaucoma and mortality: a long-term follow-up study. Acta Ophthalmol..

[3] Lütjen-Drecoll E., Shimizu T., Rohrbach M. (1986). Quantitative analysis of “plaque material” in the inner- and outer wall of Schlemm’s canal in normal and glaucomatous eyes. Exp. Eye Res..

[4] Ringvold A., Vegge T. (1971). Electron microscopy of the trabecular meshwork in eyes with exfoliation syndrome (pseudoexfoliation of the lens capsule). Virchows Arch. Abt. A Pathol. Anat..

[5] Ringvold A., Davanger M. (1977). Notes on the distribution of pseudo-exfoliation material with particular reference to the uveoscleral route of aqueous humour. Acta Ophthalmol..

[6] Schlötzer-Schrehardt U., Naumann O.G. (1995). Trabecular meshwork in pseudoexfoliation syndrome with and without open-angle glaucoma. A morphometric, and ultrastructural study. Invest. Ophthalmol. Vis. Sci..

[7] Pache M., Flammer J. (2006). A sick eye in a sick body? Systemic findings in patients with primary open-angle glaucoma. Surv. Ophthalmol..

[8] Weinreb R.N., Leung C.K.S., Crowston (2016). Primary open-angle glaucoma. Nat. Rev. Dis. Primers.

[9] Chen Y.-Y., Hu H.-Y., Chu D. (2016). Patients with primary open-angle glaucoma may develop ischemic heart disease more often than those without glaucoma: an 11-year population-based cohort study. PloS One.

[10] Lee D.J., Gómez-Marín O., Lam B.L. (2003). Glaucoma and survival. Ophthalmology.

[11] Lee A.J., Wang J.J., Kifley A. (2006). Open-angle glaucoma and cardiovascular mortality. Ophthalmology.

[12] Ringvold A. (1973). On the occurrence of pseudo-exfoliation material in extrabulbar tissue from patients with pseudo-exfoliation syndrome of the eye. Acta Ophthalmol (Copenh).

[13] Sugino T. (1990). Exfoliation materials in the skin of patients with exfoliation syndrome. Nippon. Ganka Gakkai Zasshi.

[14] Schlötzer-Schrehardt U., Koca M.R., Naumann G.O.H. (1992). Pseudoexfoliation syndrome. Ocular manifestation of a systemic disorder?. Arch. Ophthalmol..

[15] Streeten B.W., Li Z.-Y., Wallace R.N. (1992). Pseudoexfoliative fibrillopathy in visceral organs of a patient with pseudoexfoliation syndrome. Arch. Ophthalmol..

[16] Mitchell P., Wang J.J., Smith W. (1997). Association of pseudoexfoliation syndrome with increased vascular risk. Am. J. Ophthalmol..

[17] Akarsu C., Ünal B. (2005). Cerebral haemodynamics in patients with pseudoexfoliation glaucoma. Eye.

[18] Katsi V., Pavlidis A.N., Kallistratos M.S. (2013). Cardiovascular repercussions of the pseudoexfoliation syndrome. N. Am. J. Med. Sci..

[19] Siordia J.A., Franco J., Golden T.R. (2016). Ocular pseudoexfoliation syndrome linkage to cardiovascular disease. Curr. Cardiol. Rep..

[20] Chung H., Arora S., Damji K.F. (2018). Association of pseudoexfoliation syndrome with cardiovascular and cerebrovascular disease: a systemic review and meta-analysis. Can. J. Ophthalmol..

[21] Wang W., He M., Zhou M. (2014). Ocular pseudoexfoliation and vascular disease. A systematic review and meta-analysis. PloS One.

[22] Ringvold A., Blika S., Sandvik L. (1997). Pseudo-exfoliation and mortality. Acta Ophthalmol. Scand..

[23] Svensson R., Ekström C. (2014). Pseudoexfoliation and mortality: a population-based 30-year follow-up study. Acta Ophthalmol..

[24] Shrum K.R., Hattenhauer M.G., Hodge D. (2000). Cardiovascular and cerebrovascular mortality associated with ocular pseudoexfoliation. Am. J. Ophthalmol..

[25] Slettedal J.K., Sandvik L., Ringvold A. (2015). Ocular pseudoexfoliation syndrome and life span. EBioMedicine.

[26] Slettedal J.K., Sandvik L., Ringvold A. (2018). Lifespan reduction due to neoplasia is nullified by pseudoexfoliation syndrome. Heliyon.

[27] Ringvold A., Blika S., Elsås T. (1988). The Middle-Norway eye-screening study. 1. Epidemiology of the pseudo-exfoliation syndrome. Acta Ophthalmol..

[28] Ringvold A., Blika S., Elsås T. (1991). The Middle-Norway eye-screening study. 2. Prevalence of simple and capsular glaucoma. Acta Ophthalmol..

[29] French D.D., Margo C.E., Harman L.E. (2012). Ocular pseudoexfoliation and cardiovascular disease: a national cross-section comparison study. N. Am. J. Med. Sci..

[30] Scharfenberg E., Rauscher F.G., Meier P., Hasenclever D. (2019). Pseudoexfoliation syndrome: analysis of systemic comorbidities of 325 PEX-positive patients compared with 911 PEX-negative patients. Graefes Arch. Clin. Exp. Ophthalmol..

[31] Tarkkanen A., Kivelä T. (2014). Mortality in primary open-angle glaucoma and exfoliative glaucoma. Eur. J. Ophthalmol..

